# Frontotemporal correlates of impulsivity and machine learning in retired professional athletes with a history of multiple concussions

**DOI:** 10.1007/s00429-015-1012-0

**Published:** 2015-02-27

**Authors:** R. Goswami, P. Dufort, M. C. Tartaglia, R. E. Green, A. Crawley, C. H. Tator, R. Wennberg, D. J. Mikulis, M. Keightley, Karen D. Davis

**Affiliations:** 1 Canadian Sports Concussion Project, Toronto Western Hospital, Toronto, Canada; 2 Division of Brain, Imaging and Behaviour-Systems Neuroscience, Toronto Western Research Institute, Toronto Western Hospital, University Health Network, 399 Bathurst Street, Room MP14-306, Toronto, ON M5T 2S8 Canada; 3 Department of Medical Imaging, Toronto Western Hospital and University of Toronto, Toronto, Canada; 4 Tanz Centre for Research in Neurodegenerative Diseases, University of Toronto, Toronto, Canada; 5 Division of Neurology, Krembil Neuroscience Centre, Toronto, Canada; 6 Toronto Rehabilitation Institute, University Health Network, Toronto, Canada; 7 Institute of Medical Science, University of Toronto, Toronto, Canada; 8 Division of Neurosurgery, Krembil Neuroscience Centre, Toronto Western Hospital, Toronto, Canada; 9 Department of Occupational Science and Occupational Therapy, University of Toronto and Holland Bloorview Kids Rehabilitation Hospital, Toronto, Canada; 10 Department of Surgery, University of Toronto, Toronto, Canada

**Keywords:** Concussion, Impulsivity, Uncinate fasciculus, Cortical thickness, Connectivity, Machine learning

## Abstract

The frontotemporal cortical network is associated with behaviours such as impulsivity and aggression. The health of the uncinate fasciculus (UF) that connects the orbitofrontal cortex (OFC) with the anterior temporal lobe (ATL) may be a crucial determinant of behavioural regulation. Behavioural changes can emerge after repeated concussion and thus we used MRI to examine the UF and connected gray matter as it relates to impulsivity and aggression in retired professional football players who had sustained multiple concussions. Behaviourally, athletes had faster reaction times and an increased error rate on a go/no-go task, and increased aggression and mania compared to controls. MRI revealed that the athletes had (1) cortical thinning of the ATL, (2) negative correlations of OFC thickness with aggression and task errors, indicative of impulsivity, (3) negative correlations of UF axial diffusivity with error rates and aggression, and (4) elevated resting-state functional connectivity between the ATL and OFC. Using machine learning, we found that UF diffusion imaging differentiates athletes from healthy controls with significant classifiers based on UF mean and radial diffusivity showing 79–84 % sensitivity and specificity, and 0.8 areas under the ROC curves. The spatial pattern of classifier weights revealed hot spots at the orbitofrontal and temporal ends of the UF. These data implicate the UF system in the pathological outcomes of repeated concussion as they relate to impulsive behaviour. Furthermore, a support vector machine has potential utility in the general assessment and diagnosis of brain abnormalities following concussion.

## Introduction

Structures within the temporal lobe and frontal cortex form a network involved in behavioural regulation (Brothers 1990), with dense connections between the anterior temporal lobe (ATL), amygdala, hippocampus and orbitofrontal cortex (OFC) (Ghashghaei et al. 2007). Frontal and temporal neural circuitry has been implicated in the pathophysiology of behaviours such as impulsivity and aggression (Weiger and Bear 1988; Snowden et al. 2001; Winstanley et al. 2004). The uncinate fasciculus (UF) is a major white matter (WM) tract that bidirectionally connects the medial and lateral OFC with the ATL (Catani et al. 2002; Schmahmann et al. 2007), and is implicated in impulsivity, described in studies of frontotemporal dementia (Piguet et al. 2011) and in schizophrenia (Hoptman et al. 2002). Individuals having sustained concussions often exhibit behavioural changes including impulsivity, depression and aggression (Bigler 2007; Silver et al. 2009), and the UF has been implicated in concussion (Smits et al. 2011). However, a link between the UF system, impulsivity, and concussion has not been established.

Task response inhibition serves as a proxy for the behavioural attribute of impulsivity. The OFC is an important processing center with a proposed role in the inhibitory control of behaviour. OFC lesions can affect emotion, personality, and social behaviour (Kringelbach and Rolls 2004). Frontal gray matter volume is reduced in healthy subjects with high impulsivity (Matsuo et al. 2009), and response inhibition activates the lateral OFC (Horn et al. 2003). The ATL is a hub for semantic processing and social cognition (Olson et al. 2007; Wong and Gallate 2012). Both frontal and temporal lobe damage are implicated in neuropsychiatric conditions including aggression (Wong and Gallate 2012). Therefore, impaired frontotemporal function may contribute to disinhibition and other neuropsychiatric presentations that arise from functional and/or structural deficits of the UF and connected regions. Such deficits can be assessed with resting state functional connectivity, cortical thickness and diffusion tensor imaging.

Here, our first aim was to determine the relationship between response inhibition and related psychological factors to the structural and functional properties of the UF and frontotemporal gray matter in retired professional athletes with a chronic history of multiple concussions. We then used machine learning to test the predictive power of diffusion imaging metrics within the UF to discriminate these concussed athletes from controls. We hypothesized that (1) athletes would exhibit neurocognitive deficits linked with structural and functional abnormalities of the UF and surrounding gray matter, and (2) diffusion tensor WM metrics of the UF can be used in a machine learning algorithm to distinguish concussed athletes from non-concussed healthy individuals. This study has implications for our fundamental understanding of the neural underpinnings of impulsivity and their pathophysiological manifestations in concussion. The novel machine learning approach represents a first step towards a potential prognostic tool for classifying brain health in the context of multiple concussions in contact sports.

## Materials and methods

### Participants

Twenty-two retired professional athletes from the Canadian Football League (CFL), including one that played university football, were recruited through information provided by the CFL Alumni Association, and provided informed written consent to experimental procedures approved by The University Health Network Research Ethics Board. Of the 22 subjects, 19 were included in the final analysis (19 males; mean age ± SD = 50 ± 12 years with range from 30–74 years; mean education ± SD = 17 ± 1.8 years) after excluding those with contraindications to MRI or co-morbidity as listed below as exclusion criteria. The number of self-reported concussions in athletes ranged from 2 to 15. However, these numbers are likely an underestimate because of recall bias, poor concussion diagnostics in the past, and reluctance for reporting by players. Thus, we were cautious in using number of concussions as an index of severity. At least 12 out of 19 athletes reported their first concussion while in high school and 8 athletes additionally experienced 2–4 non-sports related concussions. The athletes were closely matched for age and education level to 17 healthy control subjects (17 males; mean age ± SD = 46 ± 10 years; mean education ± SD = 16 ± 1.9 years) as well as sex, with no significant differences between the groups.

Inclusion criteria for the retired athletes were as follows: under 75 years old, fluent in English, and a history of multiple concussions. Concussion exposure was based on athletes self-report. Concussion was operationally defined in accordance with the guidelines agreed upon by the International Consensus statements (McCrory et al. 2013; Tator 2013). This method of self-reported concussion has excellent test–retest reliability (intraclass correlation coefficient = 0.90) and validity (Guskiewicz et al. 2007), although anecdotal evidence suggests that professional football players may use a higher threshold for classifying an event as a concussion and thus concussions may be under-reported in this population. In addition, all players underwent a semi-structured interview to verify this information and to jog memory for any events they may not have been initially recalled. Exclusion criteria for athletes included: neurological disorders prior to concussions (e.g. seizure disorder), systemic illnesses known to affect the brain (e.g. diabetes and lupus), a history of psychotic disorder; known developmental disorders (e.g. attention deficit disorder, dyslexia), other neurological conditions (e.g. epilepsy, multiple sclerosis), and active engagement in litigation. Inclusion/exclusion criteria for control subjects were the same as above for athletes but the exception of concussion history. Control subjects were interviewed to verify they had no history of concussions or suspected concussions.

All participants completed neuropsychological assessment and neuroimaging over the course of two consecutive days. Neuropsychological assessment, including clinical interview, was completed by a psychometrist at a large, urban teaching hospital in downtown Toronto and neuroimaging was carried out within the same institution. All participants also underwent a neurological assessment by a neurologist with extensive experience in concussion.

### Neuropsychological measures

**Fig. 1:**
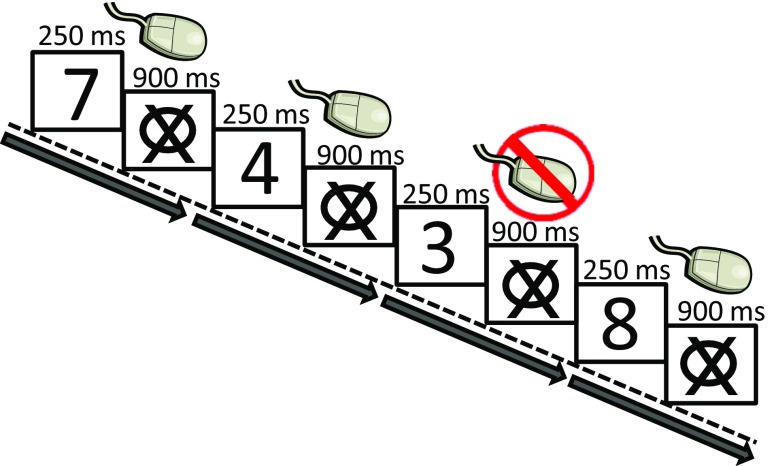
Illustration of go/no-go task. Faster reaction time and more errors were identified as an index of reduced response inhibition

All participants completed the sustained attention to response task (SART) (Robertson et al. 1997) and the Personality Assessment Inventory (PAI) as part of a larger neuropsychological test battery. The SART is a computerized go/no-go task that was designed to measure sustained attention. The SART, in its original form with random presentation of stimuli, has also been used to measure response inhibition (Helton 2009; Helton et al. 2009; O’Connell et al. 2009; Carter et al. 2013). In the SART used here, participants were presented with the digits 1–9 in random order at a rate of every 1.15 s on a computer monitor. Each digit was presented for 250 ms followed by a 900 ms inter-stimulus interval (ISI) and participants were required to respond to the appearance of each digit by clicking the mouse (“go” trials) except when they saw the number 3, where they were asked to withhold their response (“no-go” trials). Participants were instructed to respond as fast and accurately as possible. During the ISI following each digit, the visual display consisted of a ring with a diagonal cross in the middle. The task consisted of a total of 225 trials (25 of each of the 9 digits) and lasted approximately 4.3 min (Fig. 1). Errors on go/no-go tasks were used to measure response inhibition (i.e. impulsivity) (Carter et al. 2013) as was faster reaction time (Li et al. 2006; Sakai et al. 2013).

The PAI is a self-report questionnaire with good demonstrated construct validity (Morey 2007). It consists of 344 questions, with 11 clinical scales, including aggression (Morey 2007). Aggression and mania were of interest as outcome measures because of their relationship with response inhibition (Vigil-Colet and Codorniu-Raga 2004; Swann et al. 2007; Strakowski et al. 2009).

### Neuroimaging protocol

#### MRI acquisition

All subjects underwent brain imaging acquired with a 3 Tesla MRI system (GE Signa HDx, Milwaukee, WI, USA) fitted with an 8-channel phased array head coil to obtain high resolution structural images, diffusion-weighted images (DWI), and resting state functional MRI (rs-fMRI) images. A high-resolution anatomical whole-brain scan was acquired using a T1-weighted inversion recovery prepped, 3-dimensional fast spoiled gradient echo (IR-FSPGR) sequence, with the following parameters: 180 axial slices, 1 × 1 × 1-mm voxels, 256 × 256 matrix size, 25.6-cm field of view, flip angle = 15º, echo time = 3 ms, repetition time (TR) = 7.8 ms, inversion time = 450 ms. Two DWI scans were obtained with diffusion gradients applied across 60 spatial directions (*b* = 1,000 s/mm^2^) as well as 10 non-diffusion weighted (*B*_0_) scans (TR = 17,000 ms, 23-cm field of view, 96 × 96 matrix, 2.4 × 2.4 mm^2^ in-plane resolution, 2.4-mm thick axial slices). Prior to the rs-fMRI scan, participants were instructed to close their eyes, not think of anything in particular, and to not fall asleep. The scan acquisition was 5 min 8 s using T2^*^-weighted echo-planar imaging with the following parameters: TR = 2000 ms, TE = 30 ms, 64 × 64 matrix, 20-cm FOV, flip angle = 85º, 40 slices, 3.125 × 3.125 × 4 mm^3^ voxels.

#### Cortical thickness analysis

Cortical gray matter was assessed with cortical thickness analysis (CTA) using Freesurfer software v.5 (http://surfer.nmr.mgh.harvard.edu/). The details of these methods have been described previously (Dale et al. 1999; Fischl et al. 1999; Fischl and Dale 2000). Pre-processing of T1-weighted scans included transformation to Talairach space, intensity normalization, hemispheric separation, and tissue segmentation. The gray matter/WM and gray matter/CSF border were identified and modelled as surfaces. Thickness was calculated by the software as the distance between the two borders along each point of the cortex in each hemisphere. Each subject’s cortex was anatomically parcellated and each sulcus and gyrus was labelled and aligned to Freesurfer’s average surface map. A 6-mm full-width half-maximum (FWHM) Gaussian spatial smoothing kernel was applied to the dataset, and a corrected threshold of *p* < 0.05 was used based on Monte Carlo permutations with 5000 iterations using AlphaSim (http://afni.nimh.nih.gov/afni/) as previously used by our group (Moayedi et al. 2011; Erpelding et al. 2012). A general linear model (GLM) was used to assess group differences, with age included in the model as a variable of no interest. We also included impulsivity, aggression and mania measures as regressors of interest to determine the correlations between gray matter and behaviour. An ROI approach based on anatomical landmarks connecting the UF restricted the analysis to a mask consisting of the OFC areas (Brodmann areas 10, 11, 47) and temporal pole (Brodmann area 38) obtained from the cortical parcellation atlas (Brodmann) in Freesurfer.

#### Diffusion data preprocessing

Preprocessing was performed with Functional MRI of the Brain Software Library (FSL, v.4.1.8; http://www.fmrib.ox.ac.uk/fsl) (Smith et al. 2004). Affine registration transformation matrices were used to correct for eddy current and motion artefact using the FSL Diffusion Toolbox (FDT) (Jenkinson et al. 2012). The two runs of DWI data for each subject were averaged to a single volume for greater signal-to-noise ratio. Diffusion images and T1-weighted images were skull-stripped using the Brain Extraction Tool (Smith 2002). Then, the preprocessed images were fit with a diffusion tensor model using DTIFIT in the Diffusion Toolbox. Fractional anisotropy (FA) and mean diffusivity (MD) maps were created, and axial diffusivity (AD) and radial diffusivity (RD) were obtained from the images of the eigenvalues representing the magnitude of diffusion in the three principal directions [AD = *λ*_1_, RD = (*λ*_2_ + *λ*_3_)/2].

#### Probabilistic tractography

To evaluate the DTI metrics of the UF, we first delineated the UF in each subject using probabilistic tractography. To do this, we performed tractography from a seed region (i.e. a region of interest (ROI) consisting of 3–4 voxels) approximately midway between the ATL and OFC in each subject’s native diffusion space on the axial slice FA and colour orientation maps. Two exclusion masks were drawn on the sagittal slice over the anterior commissure as well as the inferior longitudinal fasciculus that enters the ATL and projects posteriorly. As a control tract, we also performed tractography on the superior longitudinal fasciculus (SLF). The seed for the SLF was drawn on the coronal section posterior to the postcentral gyrus. Exclusion masks were drawn on the internal and external capsule to avoid the corona radiata and on the inferior longitudinal fasciculus. The selection of the ROIs was overseen and inspected by two other authors who are experienced in neuroimaging and neuroanatomy.

Fiber tracking was performed using probabilistic tractography (probtrackx) in FSL based on Bayesian estimation of diffusion parameters obtained using sampling techniques (Bedpostx). Fiber tracking from each seed generated 5000 streamline samples, step length of 0.5 mm and curvature threshold of 0.2. Tractography of the left and right sides were run separately, and created probabilistic maps of the connections between the voxels in the seeds to the rest of the brain. The tractography maps were normalized to take into account the number of voxels in the seed by dividing the number of streamline samples in the voxels in the tract maps by the way-total (i.e. the total number of streamline samples not rejected from the exclusion masks). The resultant tract maps for the UF were thresholded to 20 % of the 95th percentile of the intensity values’ distribution in the voxels within the tract. To do the same for the SLF tracts, threshold of 40 % was used to exclude connections not part of the tracts and background noise. The tract maps of each subject were averaged to produce a group map.

#### Registration of tractography maps for machine learning analysis

The FSL Tract-Based Spatial Statistics (TBSS) software tool (Smith et al. 2006) was used to compensate for spatial variations in WM anatomy across subjects, producing maps of each participant’s FA, MD, AD and RD metrics spatially co-registered and aligned to a common space for direct comparison. TBSS is a widely used tool that achieves superior WM alignment in two stages. First, each subject’s FA map is nonrigidly transformed to MNI space and interpolated to a higher resolution of 1 × 1 × 1-mm in the common space. Second, a peak-finding algorithm locates the peaks in FA corresponding to the core “skeleton” of WM sheets in each patient, maps them through the nonrigid transformation, and then performs a further alignment that brings all subjects’ sheets into correspondence on a single, common skeleton. Any desired metric can then be mapped from its location on each subject’s individual WM skeleton to a corresponding location on the common skeleton for comparison across individuals. Alignment of WM core skeletons and restriction of analyses to metrics on these skeletons helps to reduce the potentially deleterious consequences of misalignment and partial volume effects.

The common skeleton and accompanying DTI metrics from each subject were restricted to the UF by transforming probabilistic tractography-based segmentations of each subject’s left and right uncinate (described above) into standardized space, averaging and thresholding them, and finally taking their intersection with the TBSS skeleton mask. In addition to the coordinates for each voxel, the 4 DTI metrics were also recorded for each voxel in each UF tract.

#### Resting state fMRI

Resting-state processing steps were performed with FSL v.4.1.8, MATLAB v.7.12.0 (MathWorks), and fMRISTAT (Worsley et al. 2002). Using FSL’s FEAT, we deleted the first 4 volumes, and performed motion correction (MCFLIRT), brain extraction (BET), and linear registration (FLIRT) between functional, T1-weighted anatomical, and standard MNI152 space (2 mm^3^ resolution) images. Removal of physiological and scanner noise was achieved using CompCor (Behzadi et al. 2007; Chai et al. 2012). This involved segmenting the T1-weighted image and registering WM and CSF partial volume maps to fMRI space. The maps were eroded (i.e. removal of voxels with low probability of being WM or CSF) by thresholding and retaining the top 198 and 20 cm^3^ of voxels with the highest probability of being WM and CSF, respectively (Chai et al. 2012). Voxels within these WM and CSF maps were masked with the 4D fMRI data and principal component analysis was run. The top five WM and CSF components as well as six motion parameters were regressed out. The data were smoothed using a 6-mm FWHM kernel and bandpass temporally filtered (0.01–0.1 Hz).

Based on the anatomical end points of the UF, we were interested in examining the functional connectivity between the ATL and OFC in athletes and controls. The ROI for the temporal lobe was based on the significant difference in cortical thickness observed in the left ATL between groups obtained from Freesurfer CTA (see “Results”). This is based on work showing abnormal resting state functional connectivity in regions with lower gray matter density and cortical thinning, suggesting morphometric effects on functional network integrity (van Tol et al. 2013). The labelled region was transformed from Talairach to MNI space and then to a binary ROI. The mean time course from the ROI was extracted and entered into a GLM. The ROI for the OFC was taken from (Kahnt et al. 2012) who performed parcellation of the human OFC into anatomical subdivisions based on resting-state connectivity with the rest of the brain. The left medial OFC (mOFC) was defined as 2-mm-diameter spheres (MNI coordinates: *x* = −17, *y* = 42, *z* = −12). In addition to using the cortical thinning finding in the left ATL and OFC coordinates as ROIs, the whole ATL region was defined by masks from cortical parcellation atlas in Freesurfer (aparc2009), as well as for the OFC (aparc2009) to examine left ATL-OFC and right ATL-OFC functional connectivity. Pearson’s correlations were calculated between each pair of ROIs and converted to Fisher Z values.

### Machine learning

A machine learning analysis was undertaken to assess the predictive power of the DTI metrics confined to the UF to (1) discriminate the athletes from controls, and (2) interrogate the spatial pattern of this predictive power over the extent of the tracts. A support vector machine (SVM) classifier (based on the LibSVM implementation) (Chang and Lin 2011) was trained on all voxels in the UF (832 left, 940 right) for each combination of the four DTI metrics and two hemispheres, for a total of eight. In each case, a leave-one-out cross-validation protocol was employed to assess accuracy in an unbiased manner, by selecting features and training the classifier on all but one of the 19 athlete and 17 control subjects and testing on the one left out. Within each fold, feature selection was performed to identify the subset of voxels most likely to yield the best results by computing an F-statistic for each voxel (Chen and Lin 2006) and retaining a specified fraction of the highest scoring voxels. Training and testing were repeated with linear, polynomial, and radial basis function (RBF) kernels. Each classifier’s performance was optimized over its hyper-parameters by executing a 2D (linear and polynomial) or 3D (RBF) grid search over the fraction of optimal features retained, the SVM cost parameter, and for the RBF kernel, the scale or gamma parameter of the kernel.

Machine learning algorithms achieve their superior accuracy through the selective application of bias in exchange for reduced variance (Hastie et al. 2009). The applied bias typically takes the form of a regularization penalty, and the various methods are distinguished by the form of the penalty they apply. Since it is generally not possible to determine which technique will perform best on a specific problem in advance, we also applied a sparsity-based logistic regression classifier (LRC) with an ElasticNet penalty (Zou and Hastie 2005), using the GLMNet coordinate descent algorithm (Friedman et al. 2010), and a structured sparse total variation LRC (Baldassarre et al. 2012) implemented using the ADMM algorithm (Boyd et al. 2011). Leave-one-out cross-validation was again used to assess accuracy, while hyper-parameter optimization over the lasso parameter was performed using the GLMNet path algorithm, and over the ElasticNet parameter and sparse total variation parameters using grid search (for further details, see Friedman et al. (2010) and Boyd et al. (2011).

To assess each classifier’s statistical significance, the entire training, cross-validation and grid search procedure was repeated 10,000 times, in each case with a different randomly permuted assignment of the concussion/control labels. The fraction of these repetitions that produced classifiers with equal or superior performance to the correct labelling was taken as the statistical significance of the classifier. Receiver operating characteristic (ROC) curves were generated using the MATLAB (MATLAB and Statistics Toolbox Release 2009b, The MathWorks, Inc., Natick, MA) PERFCURVE function.

For linear classifiers whose predicted response is formed as a linear combination of predictor variables multiplied by weight coefficients, it is common practice to visualize the resulting spatial pattern of coefficients as maps wherein each weight is overlaid on the voxel it multiplies and is colour coded to indicate its sign and magnitude (Pereira et al. 2009). However, recent work suggests that in the case of binary classifiers, visualization of the covariance between predictors and responses provides a more interpretable alternative (Haufe et al. 2014). We, therefore, examined both kinds of maps to address both possibilities. For the covariance map, the covariance between each UF tract voxel and group (−1 for controls, +1 for athletes) was computed, and statistical significance was assessed using threshold-free cluster enhancements (Smith and Nichols 2009) as implemented in FSL’s randomise function. For the linear classifier coefficient maps, the mean value of the coefficient for each voxel over 10,000 bootstrap (Efron and Tibshirani 1994) repetitions of GLMNet and sparse total variation training was computed.

### Statistical analyses

Statistical analyses were conducted using Statistical Package for the Social Sciences (SPSS v. 19). A two-sample *t* test (two-tailed) was used to test for differences between groups on the imaging data, go/no-go task and PAI outcomes. Pearson’s correlation coefficients were used for parametric correlations to obtain relationships between behavioural outcomes and the DTI metrics, cortical thickness, and functional connectivity measures. Partial correlations were calculated for DTI and cortical thickness relationships with behavioural scores to control for age at time of testing. Correlation analyses of the multiple behavioural and imaging outcomes were performed as an exploratory analysis, with the significance level set at *p* < 0.05 (two-tailed).

## Results

### Behavioural findings

**Fig. 2:**
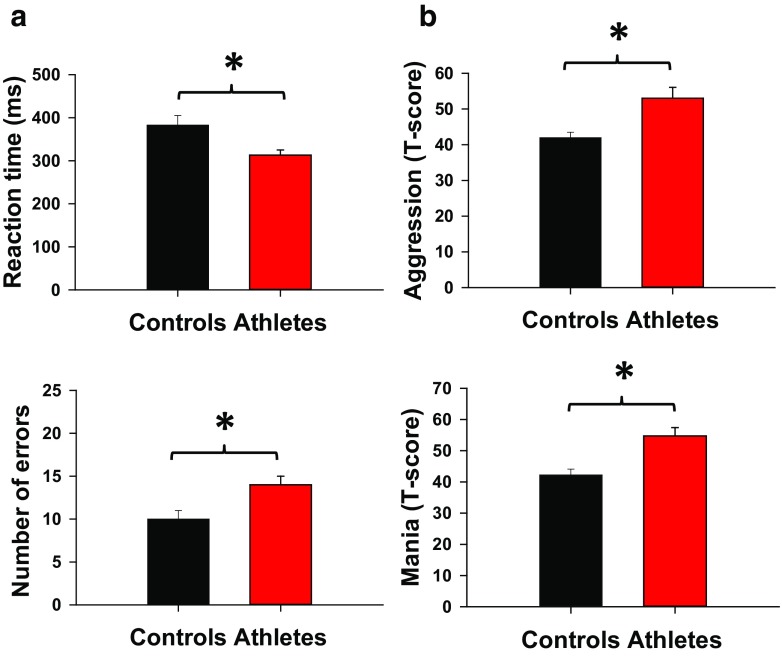
**a** go/no-go (SART) results for reaction time and number of errors (out of a possible number of 25). Athletes had faster reaction time and greater number of errors compared to controls (*p* < 0.05). **b** PAI results indicated higher aggression and mania in athletes versus controls. Data are presented as mean ± SEM; *p < 0.05

On the go/no-go task, the athlete group had faster reaction times and made more errors compared to the control group, indicative of reduced response inhibition (*p* < 0.05; Fig. 2a). Additionally, the athlete group had significantly higher mania and aggression scores on the PAI than the controls group (*p* < 0.05; Fig. 2b), although the elevated values did not reach levels high enough to be considered clinically significant.

### Cortical thinning of the ATL and OFC thickness correlates with errors and aggression

**Fig. 3:**
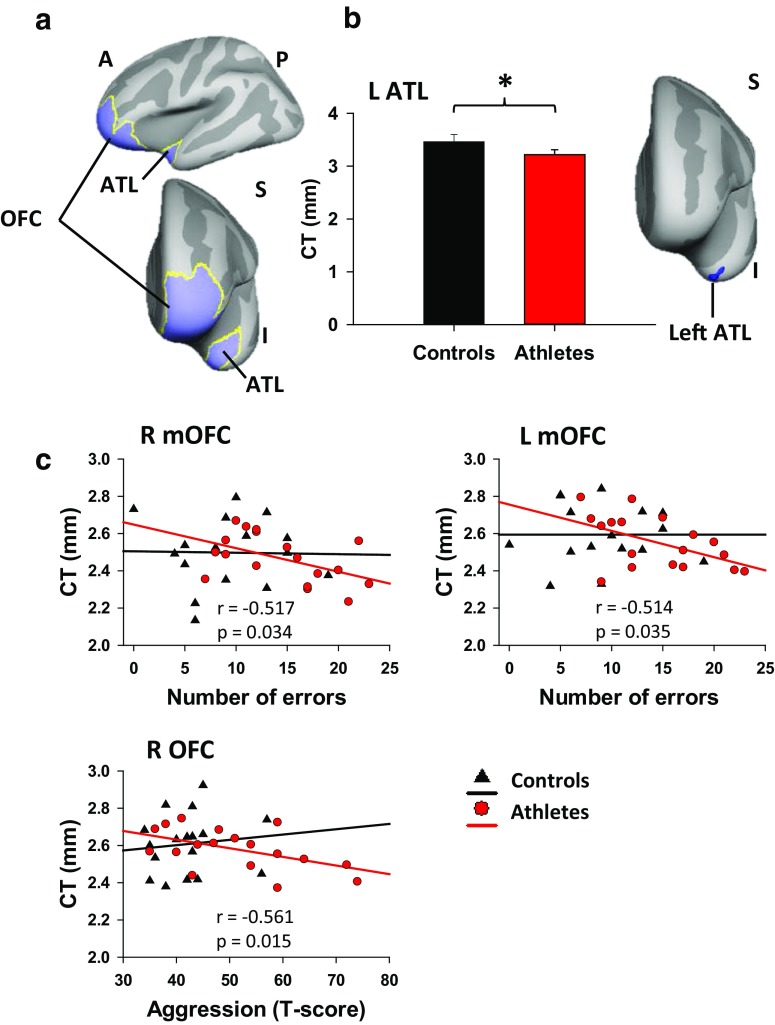
**a** Masks used in the cortical thickness analysis (CTA) were restricted to the orbitofrontal cortex (OFC; Brodmann Area 10, 11, 47) and anterior temporal lobe (ATL; Brodmann area 38) shown in *blue*. **b** Cortical thinning of the left ATL in the athletes compared to controls in a region of the ATL (*blue cluster*) (*p* < 0.05, corrected for multiple comparisons). Data are presented as mean ± SEM; *p < 0.05. **c** In the athletes, the number of go/no-go errors was correlated with cortical thickness of the right (*r* = −0.517, *p* = 0.034) and left (*r* = −0.514, *p* = 0.035) medial OFC. As well, greater aggression was correlated with reduced cortical thickness of the right OFC (*r* = −0.561, *p* = 0.015). *A* anterior, *P* posterior, *S* superior, *I* inferior, *L* left, *R* right, *mOFC* medial OFC, *CT* cortical thickness

The CTA restricted to masks of the OFC and ATL (Fig. 3a) revealed two main findings, one at the group level and the other as a function of behaviour.

At the group level, cortical thickness of the left ATL was reduced in the athletes as compared to controls (3.2 mm in athletes vs. 3.5 mm in controls; *p* < 0.05, corrected for multiple comparisons, 228 significant vertices based on a cluster threshold of 108) (Fig. 3b). However, there was no correlation between the cortical thickness of the left ATL in the athletes and SART (reaction times, errors) or PAI outcomes. The right ATL thickness though was not significantly different between groups.

To examine relationships between cortical thickness of the OFC with behaviour, we extracted values for medial and lateral OFC thickness for each subject using the Desikan-Killiany Atlas (aparc.annot), obtained from cortical parcellation in FreeSurfer. In the athletes, cortical thickness of the mOFC bilaterally was negatively correlated with SART error rate (*r* = −0.514, *p* = 0.035 for left, and *r* = −0.517, *p* = 0.034 for right) (Fig. 3c). However, there were no significant correlations between the mOFC thickness and reaction time or PAI outcomes in athletes. Cortical thickness of the right OFC (medial + lateral) was negatively correlated with aggression scores in athletes (*r* = −0.561, *p* = 0.015), but not with mania or SART outcomes (Fig. 3c). Left OFC thickness (medial + lateral) was not significantly correlated with the SART or PAI outcomes in athletes. No correlations between OFC thickness and SART or PAI variables were observed in controls.

### UF abnormalities correlate with behaviour

**Fig. 4:**
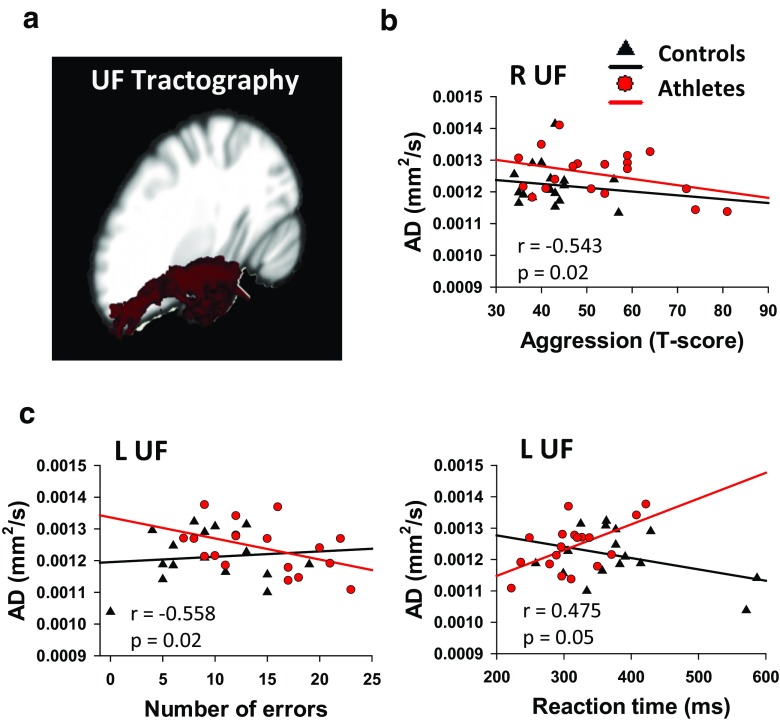
**a** Representation of probabilistic tractography group average of the uncinate fasciculus (UF). **b** Lower right UF axial diffusivity (AD) was correlated with more aggression in athletes (*r* = −0.543, *p* = 0.02). **c** Left UF AD differed between athletes and controls showing a a significant correlation with more errors (*r* = −0.558; *p* = 0.02), and a close trend for correlating with faster reaction time (*r* = 0.475; *p* = 0.05) in athletes. *L* left, *R* right

At the group level (group average UF tractography depicted graphically in Fig. 4a), there were no statistical differences for any of the 4 DTI metrics (FA, AD, RD or MD) between athletes and controls. However, AD of the UF was related to three behaviours in the athletes: (1) the right UF AD was negatively correlated with aggression (*r* = −0.543; *p* = 0.02; Fig. 4b), (2) the left UF AD was negatively correlated with the number of errors on the SART (*r* = −0.558, *p* = 0.02; Fig. 4c), and (3) there was a trend towards a positive correlation between the left UF AD with reaction time (*r* = 0.475, *p* = 0.054; Fig. 4c). No correlations between FA, RD, or MD with SART or PAI variables were observed in athletes and there were no statistically significant correlations between DTI metrics of the UF and SART or PAI outcomes in control subjects. In addition, DTI metrics of the SLF (a control tract) did not correlate with SART or PAI outcomes in athletes or controls.

### Abnormal ATL-OFC resting state functional connectivity

**Fig. 5:**
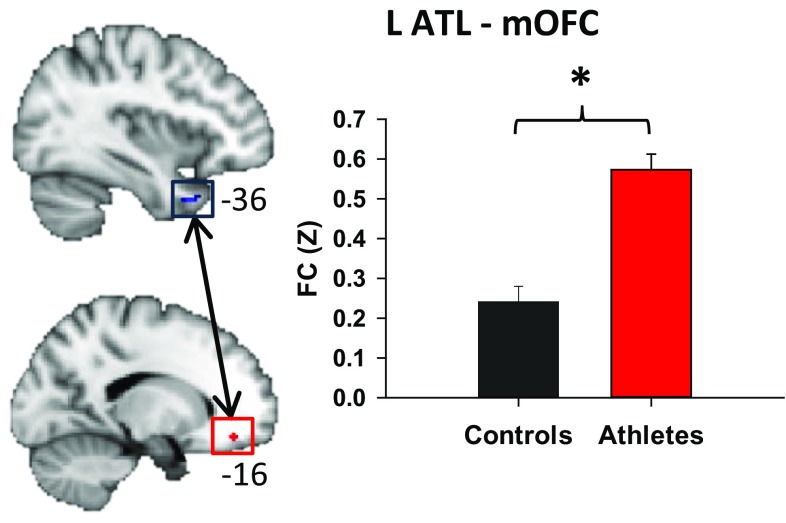
ROIs in the anterior temporal lobe (ATL) based on the region of cortical thinning observed in athletes (*in* *blue*), and orbitofrontal cortex (OFC) drawn as a 2-mm sphere (*in* *red*). On *right*, the *bar graph* depicts higher functional connectivity (FC) between the left ATL and left medial OFC in athletes versus controls (*p* < 0.05). *L* left

We determined the resting state functional connectivity of the ATL with the OFC based on the region of cortical thinning (see above) in the left ATL in athletes (Fig. 5). We found that the athletes had increased functional connectivity of this region in the left ATL with the left mOFC compared to controls (*p* < 0.05; Fig. 5). This finding was spatially specific for the region we found to show cortical thinning because there was no statistically significant difference in functional connectivity between the whole left ATL-left OFC and right ATL-right OFC, although left ATL-OFC functional connectivity was non-significantly elevated in athletes compared to controls (0.74 ± 0.04 vs. 0.49 ± 0.04, *p* = 0.088). Furthermore, the left ATL-left mOFC functional connectivity was not correlated with impulsivity or PAI variables in athletes or controls, and there were no significant correlations between the whole left ATL-OFC and right ATL-OFC functional connectivity with SART or PAI outcomes observed in either group.

### Machine learning

**Fig. 6:**
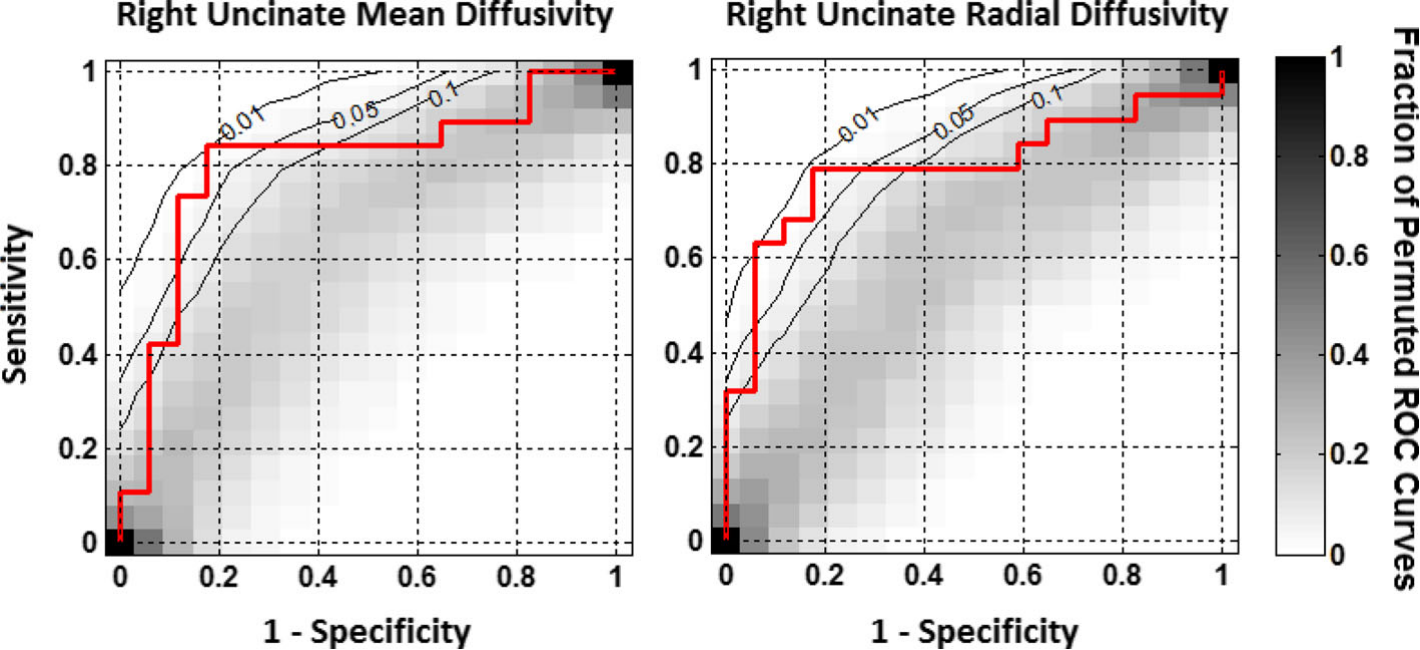
Receiver operating characteristic (ROC) curves from SVM radial basis function classifier training on the two significant DTI metrics (Table 1) measured at voxels of the right uncinate fasciculi. The actual ROC curves are depicted in *red*. Shown in *grey scale* is the proportion of 10,000 ROC curves from classifiers trained on data with the control/patient labels randomly permuted, as indicated by the *vertical bar* at *right*. The *black* *lines* are contours of this null distribution at *p* = 0.01, 0.05, and 0.1. *Red* ROC curves that penetrate the 0.05 contour are considered to be statistically significant

The results of the classifier training and testing procedure are summarized in Table 1 and illustrated in Fig. 6 for the radial basis function SVM. Out of the linear, polynomial, and RBF kernels that were tested, the RBF kernel achieved the highest accuracy and statistical significance, exceeding the performance of both the linear ElasticNet logistic regression classifier and the sparse total variation classifier. Two of the eight SVM RBF classifiers were statistically significant at *p* < 0.05. These two classifiers, trained on the MD and the RD of voxels in the right UF, achieved accuracies of 83 and 81 %, respectively. The FA in the left UF was trending toward significance with a *p* value of 0.067 and an accuracy of 78 %. Only the MD of the right UF yielded a significant result with the ElasticNet classifier, achieving an accuracy of 81 %. The sparse total variation classifier did not perform as well as the ElasticNet, and in any case achieved its highest accuracy as the total variation penalty approached zero, making it equivalent to the ElasticNet with no ridge penalty.

**Table 1:** Results of classifier training on four DTI metrics within voxels of the uncinate fasciculi

	Left uncinate			Right uncinate		
	Accuracy	*P*	AUC	Accuracy	*P*	AUC
Fractional anisotropy	0.78	0.0664	0.7183	0.64	0.3868	0.5635
Mean diffusivity	0.72	0.2032	0.6687	**0.83**	**0.0184**	**0.8050**
Axial diffusivity	0.67	0.32	0.6223	0.53	0.7884	0.2910
Radial diffusivity	0.64	0.386	0.6285	**0.81**	**0.0404**	**0.7957**

Accuracy indicates the proportion of held-out cases classified correctly by the classifier trained on the remaining cases under a leave-one-out cross-validation protocol. *P* values indicate the proportion of data sets with randomly permuted labels whose classification performance was equal or superior to the correctly labelled data. AUC indicates the area under the receiver operating characteristic (ROC) curve, a measure of performance that is agnostic to the trade-off between sensitivity and specificity

Statistically significant results are shown in bold

**Fig. 7:**
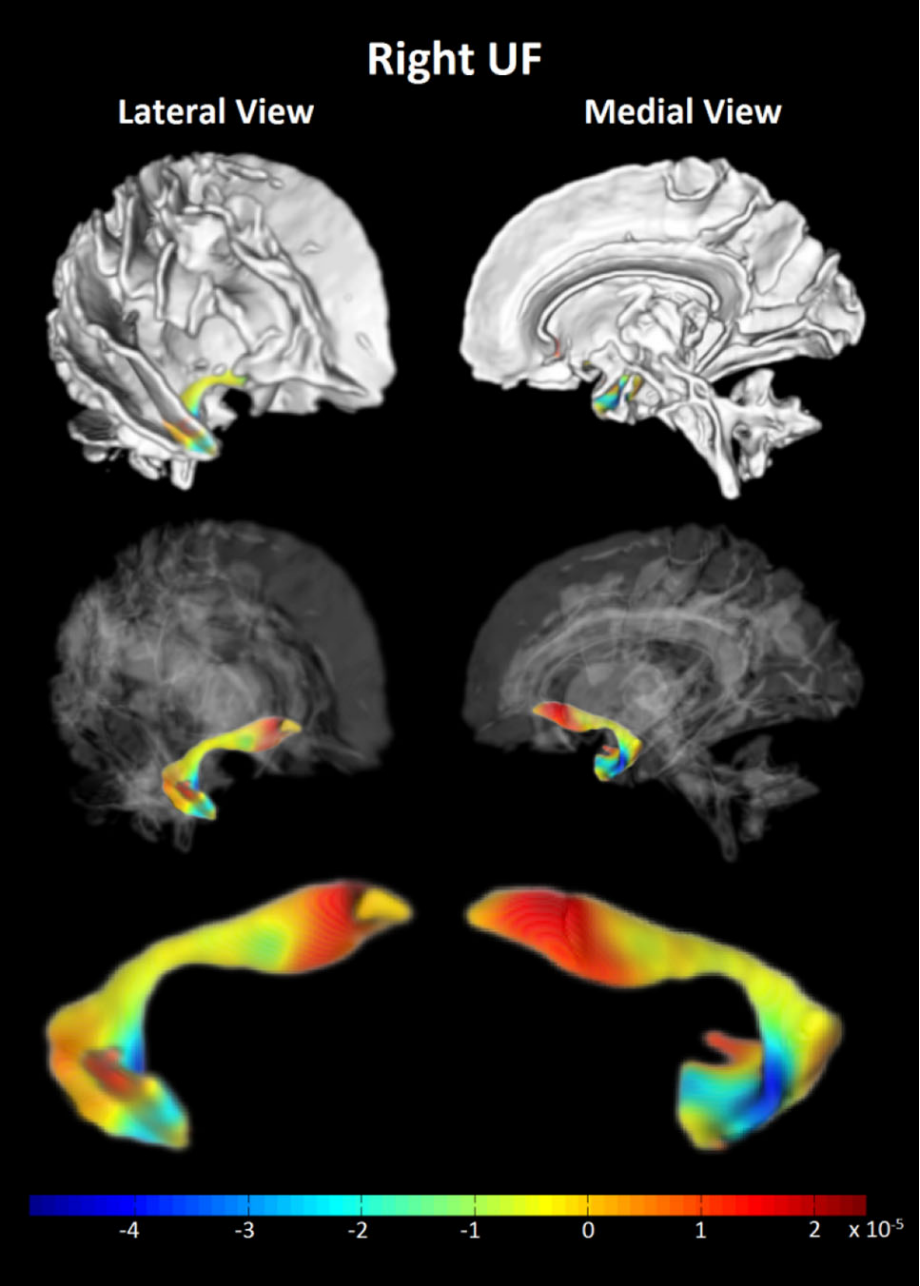
Contrasts of the spatial pattern of mean diffusivity (MD) along the right uncinate fasciculus (UF) for athletes versus controls are shown. The figure is a 3 × 2 grid of renderings of the right hemisphere white matter skeleton and UF from two different viewpoints: the lateral view of the right hemisphere from the anterior right side of the subject (*left column*), and medial view of the right hemisphere from the left of the subject (*right column*). The *top row* shows the entire right hemisphere WM skeleton as computed by TBSS, with the probabilistic tractography-based segmentation of the UF mostly obscured by other tracts. The *2nd row* makes the non-UF white matter transparent to reveal the position of the UF in context. The *bottom row* is a magnification of the UF to better reveal the pattern of covariances between the MD at each voxel and group (−1 for controls, +1 for athletes), represented by the colour. A region of high positive covariance (*red*), indicating higher MD in athletes than in controls, can be seen at the orbitofrontal end of the tract. A region of high negative covariance (*blue*), indicating lower MD in athletes versus controls, can be seen at the anterior temporal end

Since multiple classifiers were able to achieve good accuracy with statistical significance when trained on MD of several hundred voxels along the right UF, it was of interest to examine which parts of the tract were most strongly associated with differences between concussed athletes and controls. We took two approaches toward this query. First, Haufe et al. (2014) have recently presented compelling evidence arguing that spatial maps of classifier coefficients do not reveal the amount of signal (group difference in this case) present in each voxel. Indeed, they claim that these maps may reveal little of interest regarding the underlying signal. Instead, they have argued that in the case of two-group classification, the more appropriate measure is the covariance between each voxel and the grouping variable. We have, therefore, computed these quantities and rendered the associated spatial maps (see Fig. 7). Interestingly, despite the fact that none of the voxels or clusters in this map reached the level of statistical significance when controlled for multiple comparisons, our classifiers were still able to utilize this information to make accurate and statistically significant predictions of individual group membership. The spatial distribution of covariances suggests that athletes possess increased MD versus controls at the orbitofrontal end of the right UF, but decreased MD compared to controls at the anterior temporal end.

Despite the above noted objections, the rendering of classifier coefficients into spatial maps nonetheless remains a widespread practice, so it was also of interest to examine the relationship between these coefficients and the covariance map. The SVM RBF classifier attained the highest accuracy in our study, but the discriminant function of this classifier achieves its performance by combining the measurements from individual voxels in a complex nonlinear relationship that makes it difficult to assess both the sign and the magnitude of each voxel’s contribution (Pereira et al. 2009). As a tractable alternative, we therefore examined the weight coefficients computed by the ElasticNet linear classifier when trained on the MD of the right UF. Since ElasticNet coefficients have a tendency to be somewhat unstable (Hastie et al. 2009), we examined the mean of the coefficients from 10,000 bootstrap resampled training runs.

Sparse classifiers have a tendency to select only one out of a group of highly correlated predictors, setting all other coefficients in the group to zero (Tibshirani 1996). While the positive effect this strategy has on prediction accuracy is well-established, it tends to produce sparsely populated spatial coefficient maps with few, disconnected non-zero voxels sporadically distributed over the image. In this study, for example, only 8 of 941 voxels had non-zero coefficients, making the resulting Elastic Net coefficient map difficult to interpret. Nonetheless, upon comparing the values of the ElasticNet coefficients with the most significant covariances between voxels and group, we did find that they a had a close correspondence: (1) covariances and coefficients for each voxel always had the same sign; (2) their values were strongly correlated (*r* = 0.76, *p* < 10^−6^); and (3) the voxels with the greatest magnitude covariances that were statistically significant at *p* < 0.05 (uncorrected) also had the largest coefficients computed by the ElasticNet classifier.

## Discussion

This study is the first to associate diminished response inhibition (a proxy for impulsivity) in athletes with a history of multiple concussions to structural and functional abnormalities of the UF and connected gray matter. Specifically, compared to controls, retired professional football players had (1) behavioural signs of impulsivity as evidenced by reduced go/no-go response inhibition, and increased aggression and mania, (2) cortical thinning of the ATL, (3) OFC thickness that negatively correlated with task errors and aggression, (4) UF axial diffusivity that correlated with task errors and aggression, and (5) increased resting state functional connectivity between the left ATL and left mOFC. Furthermore, a machine learning algorithm trained on UF diffusion imaging metrics could differentiate the athletes from healthy controls and a spatial pattern of classifier weights revealed hot spots at the orbitofrontal and temporal ends of the UF. Taken together, our findings suggest a role for the UF frontotemporal system in impulsive and aggressive behaviour and the utility of machine learning for the diagnosis of brain injury following concussions.

Impulsivity can be defined as “the tendency to deliberate less than most people of equal ability before taking action” (Dickman 1990), often resulting in fast and inaccurate responses. Bechara et al. (2000) identified motor (or behavioural) and cognitive (or choice) impulsivity, whereby behavioural impulsivity is analogous to response inhibition and has been studied using go/no-go designs (Horn et al. 2003). Therefore, our behavioural findings that indicate impulsivity may relate to diminished response inhibition, in line with previous studies in traumatic brain injury (TBI) of increased impulsive betting behaviour (Salmond et al. 2005), and impulsivity in decision making (McHugh and Wood 2008). The go/no-go task was initially utilized to examine learning and decision making after frontal lobe damage (Drewe 1975). Lesions in the ventral medial prefrontal cortex (vmPFC), insula and anterior cingulate cortex have also been associated with impulsivity (Clark et al. 2008). Patients with OFC lesions perform worse on self-reports and cognitive/behavioural tests of impulsivity (Berlin et al. 2004). The OFC is polymodal, receiving inputs from all sensory modalities and is involved with cognitive processing and emotional and behavioural control (Stuss 2011). The OFC and ATL are directly connected through a bidirectional monosynaptic pathway via the UF, a long-range association fiber bundle (Schmahmann et al. 2007). The UF interconnections between the OFC and temporal lobe form a pathway in mediating impulsive choice, and lesions in the basolateral amygdala have been shown to disrupt the functioning of neurons in the OFC during a go/no-go task (Schoenbaum et al. 2003).

The UF is implicated in impulsive and aggressive behaviour in psychiatric disorders. Lower FA in ventromedial prefrontal WM has been shown to correlate with motor impulsivity in schizophrenia/schizoaffective disorder patients (Hoptman et al. 2004), and higher trace diffusivity in inferior frontal WM was associated with aggressiveness in schizophrenic males (Hoptman et al. 2002). While conventional neuroimaging methods often underestimate the extent of damage after concussions, DTI techniques in mild TBI (mTBI) have revealed neuropathological disruptions in widespread WM tracts including the corpus callosum, SLF, cingulum, fornix, and UF (Kraus et al. 2007; Niogi et al. 2008). Niogi and colleagues (2008) showed that DTI abnormalities in the UF correlated with performance on memory tasks. As well, UF structure predicts behavioural deficits in paediatric TBI (Johnson et al. 2011). However, UF WM changes in concussions and the relationship to impulsivity has not been previously investigated. Our findings of correlations between AD and impulsivity and aggression suggest aberrant frontotemporal structural connectivity in athletes with a history of concussions. Most studies of DTI in concussion have focused on changes in FA (for reviews on neuroimaging of mTBI see Shenton et al. 2012; Bigler 2013), and fewer have reported AD, and the latter may be more representative of axonal pathology (Song et al. 2003). Importantly, AD did not correlate with impulsivity in the SLF, a WM tract also vulnerable to concussion injury indicating specificity to the UF. Our ROI approach provides an advantage for detecting subtle WM changes in concussed individuals whereas recent work shows that whole-brain analysis does not detect WM abnormalities in acute mTBI (Ilvesmaki et al. 2014). Our findings indicate that dysfunction in this circuitry between the frontal and temporal cortices plays a role in impulsive/aggressive behaviour.

The frontal cortex and temporal lobe are often damaged in focal and diffuse brain injury (Bigler 2007; Zappala et al. 2012). We showed significant cortical thinning in the left ATL in athletes compared to controls. This is a novel finding in contrast with previous observations of medial temporal lobe damage in mTBI (Umile et al. 2002). Reduced gray matter volume in chronic mTBI has been found in frontal and temporal cortices (Gale et al. 2005). In mild and moderate/severe brain injury patients, Newcombe et al. (2011) reported a higher impulsivity index assessed by the Cambridge Gambling Task that was associated with an increase in the DTI apparent diffusion coefficient in the OFC, insula and caudate. Abnormalities in brain structure linked with impulsivity have also been noted in bipolar disorder. Reduced gray matter in prefrontal and medial temporal lobe has been associated with greater impulsivity assessed by the Barratt Impulsivity Scale (Soloff et al. 2008). In the present study, cortical thinning in the left ATL did not correlate with impulsivity, suggestive of involvement of the ATL in other putative functions (Bi et al. 2011). However, the negative correlations between OFC thickness with task errors and aggression reported here are in line with evidence of OFC damage and impulsivity (Berlin et al. 2004; Wood and Thomas 2013) and frontal lobe involvement in anger and aggression (Potegal 2012).

Our finding of enhanced functional connectivity between the area of cortical thinning in the left ATL and the left mOFC in athletes links gray matter abnormalities with altered functional regulation, possibly due to a more temporally coherent pattern of activity in the frontotemporal connectivity. This connectivity increase in athletes may also be due to underlying changes in WM as well as decreased variability in functional connectivity. Measurement of functional connectivity variability in this population is an area for future study since intrinsic inter-subject variability in functional connectivity and associated individual performance variability across various cognitive domains exists (Mueller et al. 2013).

The application of machine learning to DTI metrics in concussed athletes represents a novel approach towards damage prediction. Although Aribasala et al. (2010) achieved good accuracy using SVM with MD, they used only 16 predictors comprised MD averages over large ROIs. Hellyer et al. (2013) applied SVM to individual voxels in the entire TBSS WM skeleton and achieved very high accuracy, but subjects in their classifier study included those with severe TBI and all had microbleeds, making the classification task arguably easier. Nonetheless, in agreement with our results, they did find that their classifier exploited decreased values of DTI metrics in some regions versus increased values in other regions rather than homogenous increases or decreases over the whole cortex. Our results of a heterogeneous distribution of MD/RD in the UF (Fig. 7) may reflect the underlying anatomy wherein the temporal segment originates from the anterior temporal convolutions (area 20, 38), the uncus (area 35) and cortical nuclei of the amygdala (area 28, 34, 36) (Ebeling and von Cramon 1992). Cell bodies reside in the temporal segment and the tract runs upward over the lateral nucleus of the amygdala, through the limen insula, and then passes through the extreme and external capsule. The frontal fan-shaped portion passes in the orbital region (area 11, 47), which branches and terminates in the lateral OFC and frontal pole (Catani et al. 2002; Von Der Heide et al. 2013). Zhou et al. (2013) also reported the training of highly accurate classifiers for discriminating mTBI patients from controls, but employed a wide array of imaging modalities in their classifier concurrently. To the best of our knowledge, ours is the first study to investigate the predictive power of DTI metrics in voxels of a specific WM tract for discriminating concussed patients from controls. Our findings, together with the other studies cited, indicate that machine learning methods can reveal significant group differences that are undetectable with traditional, univariate approaches.

Limitations of our study include a cohort effect with possible self-selection to participate in the study as well as restrictions on generalizability of the findings to others in this unique cohort of individuals with very high concussion and sub-concussion exposure, but not to people with a single or a few concussions. Future studies with a greater sample size will allow for a larger network analysis of other brain regions as well as address mechanistic issues of the relationship between structure, atrophy, connectivity and impulsivity. Another limitation pertains to interpretation of the SART data as relating to inhibitory control. Inhibitory control plays an important role in normal cognition and in clinical disorders and thus we used the go-no/go task to gain insight into participants’ abilities to suppress a pre-potent response. In this task, an inter-relationship between faster speed and accurate inhibition may exist and it is thus possible that the increased error rate in the athletes may have been due in part to faster responding. However, in addition to fast responding, high error rates may arise from reduced response inhibition. Previous work suggests that impulsive individuals appear unable to inhibit pre-potent responses because their inhibitory responses are slowed and not because their pre-potent responses are remarkably faster (Logan et al. 1997). We acknowledge that the source of errors may have been contaminated with factors other than reduced response inhibition. Thus, future studies could investigate the generalizability of our findings through other measures of impulsivity and response inhibition.

Finally, our machine learning approach is novel in its application to concussion and provides a new avenue with which to further study brain abnormalities in concussed individuals. This represents a step towards development of a tool with the potential for clinical utility to predict recovery and assess clinical treatment.

In summary, we have shown heightened impulsive/aggressive behaviours in concussed athletes, with associated microstructural changes in the UF, in addition to cortical thinning in the ATL, OFC thickness that negatively correlates with higher aggression, and enhanced resting state connectivity between the left ATL and left OFC. Machine learning on DTI metrics is a useful technique to discriminate and potentially diagnose concussion. Future use of these strategies may also be useful for examining the brain structure and function related to cognitive changes in the progressive development of neuropathological changes in concussed individuals (Hazrati et al. 2013; Tator 2014).
